# Airborne Transmission Route of COVID-19: Why 2 Meters/6 Feet of Inter-Personal Distance Could Not Be Enough

**DOI:** 10.3390/ijerph17082932

**Published:** 2020-04-23

**Authors:** Leonardo Setti, Fabrizio Passarini, Gianluigi De Gennaro, Pierluigi Barbieri, Maria Grazia Perrone, Massimo Borelli, Jolanda Palmisani, Alessia Di Gilio, Prisco Piscitelli, Alessandro Miani

**Affiliations:** 1 Department of Industrial Chemistry, University of Bologna, Viale del Risorgimento 4, 40136 Bologna, Italy; 2 Interdepartmental Centre for Industrial Research “Renewable Sources, Environment, Blue Growth, Energy”, University of Bologna, 47921 Rimini, Italy; fabrizio.passarini@unibo.it; 3 Department of Biology, University “Aldo Moro” of Bari, 70121 Bari, Italy; gianluigi.degennaro@uniba.it (G.D.G.); jolanda.palmisani@uniba.it (J.P.); alessia.digilio@uniba.it (A.D.G.); 4 Department of Chemical and Pharmaceutical Sciences, University of Trieste, 34127 Trieste, Italy; barbierp@units.it; 5 Environmental Research Division, TCR TECORA, 20094 Milan, Italy; mariagrazia.perrone@tcrtecora.com; 6 Department of Life Sciences, University of Trieste, 34127 Trieste, Italy; borelli@units.it; 7 UNESCO Chair on Health Education and Sustainable Development, University of Naples Federico II, 80131 Naples, Italy; priscofreedom@hotmail.com; 8 Italian Society of Environmental Medicine (SIMA), 20149 Milan, Italy; alessandro.miani@unimi.it; 9 Department of Environmental Sciences and Policy, University of Milan, 20133 Milan, Italy

**Keywords:** COVID-19, airborne transmission, social distancing, droplets, persistence

## Abstract

The COVID-19 pandemic caused the shutdown of entire nations all over the world. In addition to mobility restrictions of people, the World Health Organization and the Governments have prescribed maintaining an inter-personal distance of 1.5 or 2 m (about 6 feet) from each other in order to minimize the risk of contagion through the droplets that we usually disseminate around us from nose and mouth. However, recently published studies support the hypothesis of virus transmission over a distance of 2 m from an infected person. Researchers have proved the higher aerosol and surface stability of SARS-COV-2 as compared with SARS-COV-1 (with the virus remaining viable and infectious in aerosol for hours) and that airborne transmission of SARS-CoV can occur besides close-distance contacts. Indeed, there is reasonable evidence about the possibility of SARS-COV-2 airborne transmission due to its persistence into aerosol droplets in a viable and infectious form. Based on the available knowledge and epidemiological observations, it is plausible that small particles containing the virus may diffuse in indoor environments covering distances up to 10 m from the emission sources, thus representing a kind of aerosol transmission. On-field studies carried out inside Wuhan Hospitals showed the presence of SARS-COV-2 RNA in air samples collected in the hospitals and also in the surroundings, leading to the conclusion that the airborne route has to be considered an important pathway for viral diffusion. Similar findings are reported in analyses concerning air samples collected at the Nebraska University Hospital. On March 16th, we have released a Position Paper emphasizing the airborne route as a possible additional factor for interpreting the anomalous COVID-19 outbreaks in northern Italy, ranked as one of the most polluted areas in Europe and characterized by high particulate matter (PM) concentrations. The available information on the SARS-COV-2 spreading supports the hypothesis of airborne diffusion of infected droplets from person to person at a distance greater than two meters (6 feet). The inter-personal distance of 2 m can be reasonably considered as an effective protection only if everybody wears face masks in daily life activities.

## 1. COVID-19: What Evidence Is There about a Possible Airborne Route of Transmission?

At the end of nineteenth century, Carl Flugge hypothesized that micro-organisms were diffused from a person to another through the droplets emitted from nose and mouth at a maximum distance of 2 m. Between 1934 and 1955, William Firth Wells theorized that droplet nuclei are sufficiently small to remain suspended in the air for a long time and still be infective. Recently published studies support the hypothesis of virus transmission over a distance of 2 m from an infected person. To date, an in-depth knowledge of mechanisms underlying the transmission process is a priority both to predict the further development of the pandemic and to prevent possible outbreak relapses caused by SARS-COV-2, a virus which still needs a better understanding of its pathogenic mechanisms. 

Current assumptions on COVID-19 transmission processes differ in the models simulating the fate of the virus into the air. Some of the assumptions are supported by experimental data, while others still need to be more deeply explored. However, in a recent paper by *van Doremalen* (2020), it has been demonstrated the higher aerosol and surface stability of SARS-COV-2 as compared with SARS-COV-1, with the virus remaining viable and infectious in aerosol for hours [1]. Although these findings come from laboratory experiments, they are enough to support the airborne transmission of SARS-COV-2 due to its persistence into aerosol droplets in a viable and infectious form. Based on the available knowledge, Morawska and Cao (2020) highlighted that small particles with viral content may travel in indoor environments, covering distances up to 10 meters starting from the emission sources, thus activating aerosol transmission [2]. Similarly, Paules et al. (2020) recently pointed out that the airborne transmission of SARS-COV-2 may also occur besides close distance contacts [3]. Both experimental and computational fluid dynamic approaches support these assumptions. 

Concerning this, Sharfman et al. had already elucidated in 2016 the fragmentation processes of muco-salivary fluids once emitted through human sneeze and coughs. Fast photography application allowed them to show the physics behind size distribution of droplets and to determine the distance that viral emissions can reach [4]. Indeed, Asadi et al. (2019) paid more attention to the particle’s number and to the size distribution of aerosol emissions occurring during human speech, discovering a high variability among individuals [5]. A further instrumental approach for droplet visualization in the exhalations produced during ordinary speaking was provided by Anfinrud et al. (2020), and the protective effect of face masks was evaluated as well [6]. Moreover, the recent insight by Bourouiba (2020) addressed the potential long distances covered by SARS-COV-2 through cough and sneeze, showing how the current knowledge on the size and number distributions of human aerosol emissions leads to consider the traditional cut-off of 5 mm used to discriminate small droplets from large ones as outdated. The same study has also highlighted that small droplets, directly emitted during a sneeze, may reach distances of 7–8 m [7].

Hosotani et al. (2013) examined Computational Fluid Dynamic (CFD) simulations concerning the spread of virus bearing droplets inside selected indoor environments. By taking into account size distribution and time, CFD, simulations allowed the authors to derive the permanence times of influenza droplet clouds in public metro transportation [8]. Updated CFD simulations, describing the spread of SARS-COV-2 emissions by an infected shopper in indoor spaces such as a supermarket, have been provided by the Kyoto Institute of Technology (simulator available online), based on the studies carried out by Iwasaki, Yamakawa and Matsuno [9].

As a result, it has been highlighted how the distance of 1–2 m among persons is not enough to safeguard from contagion risks in the absence of face masks [10]. Moreover, on the basis of criteria for outdoor social distancing among runners and bikers derived by CFD simulations, Bloken et al. (2020) pointed out the need for taking into account the potential effect of winds and different droplet size distributions, along with the position of the infected emitters with respect to the susceptible receptors [11]. A relevant issue for the assessment of the significance of airborne viral transmission is the identification of virus viability conditions in the atmosphere. Yang et al., in two different papers (published in 2011 and 2012), investigated the association between influenza A virus viability and environmental factors such as relative humidity (RH) and aerosol composition (salt, proteins, mucus), underlining the potential impact of RH on virus survival in its aerosol carrier [12,13]. As already mentioned, SARS-COV-2 and SARS-CoV-1 aerosols were prepared under laboratory conditions and deeply investigated by Van Doremalen et al. (2020), showing that the SARS-COV-2 virus may remain infectious into aerosol droplets for hours, despite the need for acquiring further knowledge on airborne virus viability [1].

A number of studies investigated the interaction between airborne particles and viruses. The study carried out by Ye et al. (2016) demonstrated that Respiratory Syncytial Virus (RSV) infection, responsible for pneumonia in children due to penetration in the deepest parts of respiratory apparatus, was boosted by particle-based transport [14]. A positive correlation between the infection rate and the particulate matter fractions PM2.5 (r = 0.446, *p* < 0.001) and PM10 (r = 0.397, *p* < 0.001) was shown. Similar findings were reported in the paper by Cheng et al. (2017) by matching data on a daily number of measles cases and PM2.5 concentrations observed in 21 Chinese cities during October 2013 and December 2014 [15]. The authors highlighted that an increase in PM2.5 equal to 10 µg/m^3^ was significantly associated with a higher incidence of measles, providing the final recommendation to foster PM reduction strategies in order to slow down the infection diffusion rate. Following these preliminary findings, Peng et al. in 2020 provided additional evidence on the interaction between particles and viruses, demonstrating that high PM concentration levels significantly affected the measles spread in Lanzhou (China) [16]. Moreover, these authors suggested reducing PM concentration levels with the purpose to lower the potential risks of measles outbreaks in the exposed population. 

Furthermore, specific analyses were performed on the microbiome adsorbed onto airborne particulate matter (PM2.5 and PM10) over a period of 6 months between 2012 and 2013 in Beijing city, showing the variability of microbiome composition depending on the examined month [17]. More specifically, the analysis of relative abundance distribution of the microbiome onto PM over the time showed the highest abundance of viruses in January and February, simultaneously with the occurrence of severe PM pollution events. Other studies dealing with the association between PM and infectious disease incidence (e.g., influenza, haemorrhagic fever with renal syndrome) confirmed that the inhalation of particles may promote virus penetration into the deepest parts of respiratory apparatus, thus enhancing the induction of infections [18].

The current knowledge on coalescence phenomena suggests that the stabilization of aerosols into the atmosphere requires specific conditions of temperature (0–5 °C) and relative humidity (90–100%). It is generally assumed that the inactivation rate of viruses into the atmosphere is promoted by an increase in temperature and solar radiation. On the contrary, high levels of relative humidity may play a key role in viral spread, resulting in an increased virulence. In this regard, Ficetola et al. (2020) recently showed that the spread of SARS-COV-2 peaked in temperate regions of the Northern Hemisphere with a mean temperature of 5 °C and a mean humidity of 0.6–1.0 kPa, while it decreased in warmer and colder regions [19]. Other studies addressed the issue of viral diffusion paying more attention to the long-range transport of pathogens associated with air masses displacement. With specific regard to viruses, a positive correlation between virus deposition rates and organic aerosols was demonstrated by Reche et al. (2018) leading the authors to the conclusion that, if compared to bacteria, virus spread could be even further [20]. Dust storms have been considered very effective in the spread of viruses. Ambient influenza A virus spread was shown to be promoted during the Asian dust days when dust particles levels were significantly higher than during the average days [21]. Another relevant case was the H5N2 avian influenza diffusion across the USA in 2015, from Iowa to the neighbour states, attributed to the transboundary spread of airborne virus carried by fine PM [22]. The long-range transport of airborne bacteria and viruses was associated with the formation of aggregates with both primary and secondary particles. 

Indeed, very long distances could be covered, particularly when transport occurs through the stratosphere. Unlike the troposphere, where particles may be removed via precipitation, the residence time of particles with viruses into the stratosphere is estimated to be a few days, or even some months [23]. It has also been highlighted that dust particles may protect the pathogens adsorbed onto their surface, allowing them to be less exposed to both radiation and toxic gases [24]. If the stratospheric may appear an extreme event, on-field studies from the outbreak of the epidemic may provide reliable support for the airborne route of the contagion. The presence of SARS-COV-2 on airborne particles was confirmed by on-field studies carried out by Liu et al. (2020) inside Wuhan Hospitals. SARS-COV-2 RNA was detected in air samples collected inside the hospitals and in the surroundings, leading the authors to the conclusion that the airborne route has to be considered an important pathway for contamination [25]. Similar findings are reported in the study of Santarpia et al., where the presence of SARS-COV-2 was detected in air samples collected at the Nebraska University Hospital [26]. On the contrary, airborne SARS-COV-2 presence was not confirmed in the study by Ong et al. (2020) but this negative evidence is likely to be related to a poor air sampling experimental design [27]. 

With regard to particles’ role in the viral diffusion process, we have produced a position paper emphasizing the airborne route as a possible additional factor for interpreting the anomalous outbreaks in northern Italy, ranked as one of the most polluted areas in Europe characterized by high PM concentrations [28,29]. A research carried out in the U.S. by Xiao et al. seems to confirm an association between increases in particulate matter concentration and mortality rates due to COVID-19 [30]. The hypothesis is that aerosol droplets emitted by infected persons during sneezing, coughing or simply talking are stabilized in the air through the coalescence with PM at high concentrations and under conditions of atmospheric stability. 

In addition, to test the presence of SARS-COV2 on PM, further studies should include the real-time assessment of the vitality of the virus as well as its potential virulence when adsorbed on particulate matter. Small droplets of a virus are meant, under normal conditions of clean air and atmospheric turbulence, to undergo evaporation and/or to disperse quickly into the atmosphere. When conditions of atmospheric stability and high PM concentrations occur, viruses may create clusters with the particles and, by reducing their diffusion coefficient, enhance both their residence time and abundance into the atmosphere. Finally, it must be said that air pollution could also influence the COVID-19 outbreak progression by increasing the host susceptibility to viral infection by independently increasing the baseline risk of cardiovascular events and complications, chronic obstructive pulmonary diseases (COPD), and other conditions that are known to increase the severity of the infection. 

## 2. Beyond 2 m/6 Feet

In conclusion, the available information about the SARS-COV-2 spreading worldwide supports the hypothesis of a model of airborne droplets from person to person at a distance greater than two meters. The potential coalescence phenomena occurring between droplets’ nuclei and particulate matter are considered plausible, especially under favourable environmental conditions (e.g., low temperature and high relative humidity levels), allowing droplet nuclei to be stabilized. On the basis of the above discussed evidence, it is reasonable to describe this viral transmission model as a ‘super-spread event’, as demonstrated by the high estimated values of the basic reproductive number (R0) in northern Italy at the early stages of the pandemic (e.g., February 2020). Therefore, the mandatory adoption of face masks would be desirable during both the lockdown and phase 2, when the progressive return to normal life is expected. Face masks represent a barrier useful to contain viral droplets nuclei exhaled by infected people as well as adequate to reduce probability of inhalation of such droplets by the surrounding healthy persons. Moreover, more extensive distancing measures (distance among persons up to 10 m) should be adopted inside indoor environments when face masks are not used. In the case of the common use of face masks, the distance among persons could be reduced to 2 m. Most common face masks, covering the human upper airways, do not allow ACE2 proteins placed in the mucous membranes of nose and mouth to enter in contact with the virus. In outdoor conditions, droplets nuclei are subjected to higher dispersion in the atmosphere—even if aggregated to particulate matter—and, in the absence of face mask, a low risk of contagion is guaranteed even for inter-personal distance shorter than 10 meters. Finally, the scientific evidence about the association among PM levels and SARS-COV-2 spreading points out the opportunity to strengthen strategies for the reduction of PM emitted by anthropogenic sources, as well as to mitigate citizens’ exposure to PM and uncontrolled aerosols. 
